# RheumaPain: a multimodal biosignal dataset for objective pain assessment in pediatric rheumatic diseases

**DOI:** 10.1016/j.dib.2026.113131

**Published:** 2026-08-03

**Authors:** Elif Yıldırım, Fatma Patlar Akbulut

**Affiliations:** a Department of Computer Engineering, Istanbul Technical University, Istanbul, Turkey; b Department of Software Engineering, Istanbul Kültür University, Istanbul, Turkey

**Keywords:** Pain assessment, Pain classification, Signal processing, Physiological signal dataset, Multimodal signals, Biosignal analysis, Pain intensity estimation

## Abstract

Pain assessment continues to pose a considerable challenge owing to the subjective characteristics of self-reports and the fluctuating intensity of pain. We introduce RheumaPain, an innovative multimodal dataset aimed at reconciling subjective feedback with physiological responses. The dataset contains synchronized physiological recordings from 42 pediatric patients diagnosed with rheumatic diseases (31 female and 11 male; mean age: 13 years) during physiotherapy sessions. We measured Electrodermal Activity (EDA), Blood Volume Pulse (BVP), triaxial Acceleration (ACC), and Skin Temperature (TEMP) with a wristworn wearable device. Signals are in sync with pain intensity scores of the Wong–Baker Faces Pain Scale which shows the difference between resting and exercising phases. The RheumaPain dataset comprises approximately 5.5 h of synchronized multimodal physiological recordings. RheumaPain also has demographic metadata and diagnostic categories of various rheumatic diseases like JIA, SLE, and JDM. It provides a unique basis for personalized pain management and the creation of objective clinical tools in pediatric care.

Specifications Table

**Table:** 

Subject	Health and medical sciences Computer Science AI in Healthcare Data Science
Specific subject area	Pain recognition, Biosignal classification, Pain analysis, Signal processing, Rheumatic Disease analysis, Pain in rheumatic diseases
Type of data	Raw, Processed
Data collection	In this project, the signal data is collected with the E4 Empatica wristband. This wristband includes different kinds of physiological signals including HR, EDA, ACC and BVP. Data collection varies for each patients’ duration of treatment, it took 8.59 min on average.
Data source location	Institution: Istanbul Kultur University, Department of Computer Engineering City/Town/Region: Istanbul, Bakırköy, Ataköy Country: Turkey Latitude and longitude (and GPS coordinates, if possible) for collected samples/data: 41° 5 7.3284′’ N 29° 2 22.3836 ’ E
Data accessibility	Repository name: Mendeley Data Data identification number: Direct URL to data: https://data.mendeley.com/datasets/y6pgjdj22f/3
Related research article	*None*

## 1 Value of the Data

• The RheumaPain dataset is a unique tool for examining subjective feedback from patients together with biosignal data, making pain analysis in rheumatic diseases more measurable. The dataset consists of biosignal recordings from 42 underage patients along with self-reported pain scale assessments collected during both resting and exercise phases. With this data, it becomes possible to understand the dynamics of pain in both rheumatology and pediatric care.
• It combines objective data obtained from biosignals collected using the Empatica E4 device with subjective reports of patients’ experienced pain levels, assessed using Wong-Baker Faces Pain Scale, which is one of the most commonly used pain scales for children in the literature. The dataset also includes demographic information such as gender, age, and the diagnosed rheumatic disease of each patient. Also, pain levels during exercise and resting phases are recorded separately.
• With this dataset, researchers get the opportunity for modelling the behaviour of pain in children, analysing the pain levels of patients with rheumatic diseases and can develop strategies for pain management.
• Researchers from different fields such as artificial intelligence, rheumatology, and biomedical engineering are able to work with this dataset.
• Developing AI-based systems to estimate pain levels from biosignal data is especially valuable for patients who have difficulty expressing their pain verbally. In this context, the focus on pediatric patients is particularly beneficial, as it enables researchers to better understand and evaluate pain experiences in children.

## 2 Background

Pain is a subjective experience that can be perceived differently by each patient, which makes pain assessment inherently challenging. Traditionally, pain assessment strategies have mainly relied on self-reporting methods, and various pain scales have been developed for this purpose [[1], [2], [3], [4], [5]]. With the advancement of artificial intelligence–based approaches, biosignal data have increasingly been analyzed alongside patients’ self-reports to provide a more objective understanding of pain. Several datasets have been introduced in the literature focusing on different contexts, such as neonatal pain assessment[6], cancer-related pain[7], and experimentally induced pain[8]. Prior to the development of the present dataset, we introduced the **PhysioPain** dataset [9], which includes multimodal data collected from 93 adult participants. Besides the physiological signals and self-reported pain intensity scores, the dataset also includes demographic information, as it is aimed to provide a comprehensive resource for pain-related research.

Assessing pain in patients with rheumatic diseases presents additional challenges compared to many other types of pain. In many rheumatic conditions, patients may not experience significant pain while at rest, and pain often emerges or intensifies during movement. Although this may seem beneficial at first, reduced movement can worsen inflammation over time, leading to further limitations in mobility and negatively affecting patients’ overall quality of life [10].

RheumaPain dataset is created to solve the challenges above by combining self-reported data and biosignal data of pediatric patients in the rheumatology department. When patients are in treatment with physiotherapists, self-reported data is collected using pain scale during both exercise and resting phases, while their biosignal data is recorded using the wristband. Also, demographic information of the patients are added to the dataset, with the type of rheumatic disease information. This structure creates a comprehensive resource for studying the complex and evolving nature of pain. By combining subjective reports with objective biosignal data, the dataset supports more accurate pain assessment, classification, and the development of personalized treatment strategies.

## 3 Data Description

In total, the dataset includes 80,182 biosignal samples (at 4 Hz) were collected from patients using the wristband. In addition to physiological measurements, pain levels of the patients including both exercise and resting phases are included.

**Fig. 1:**
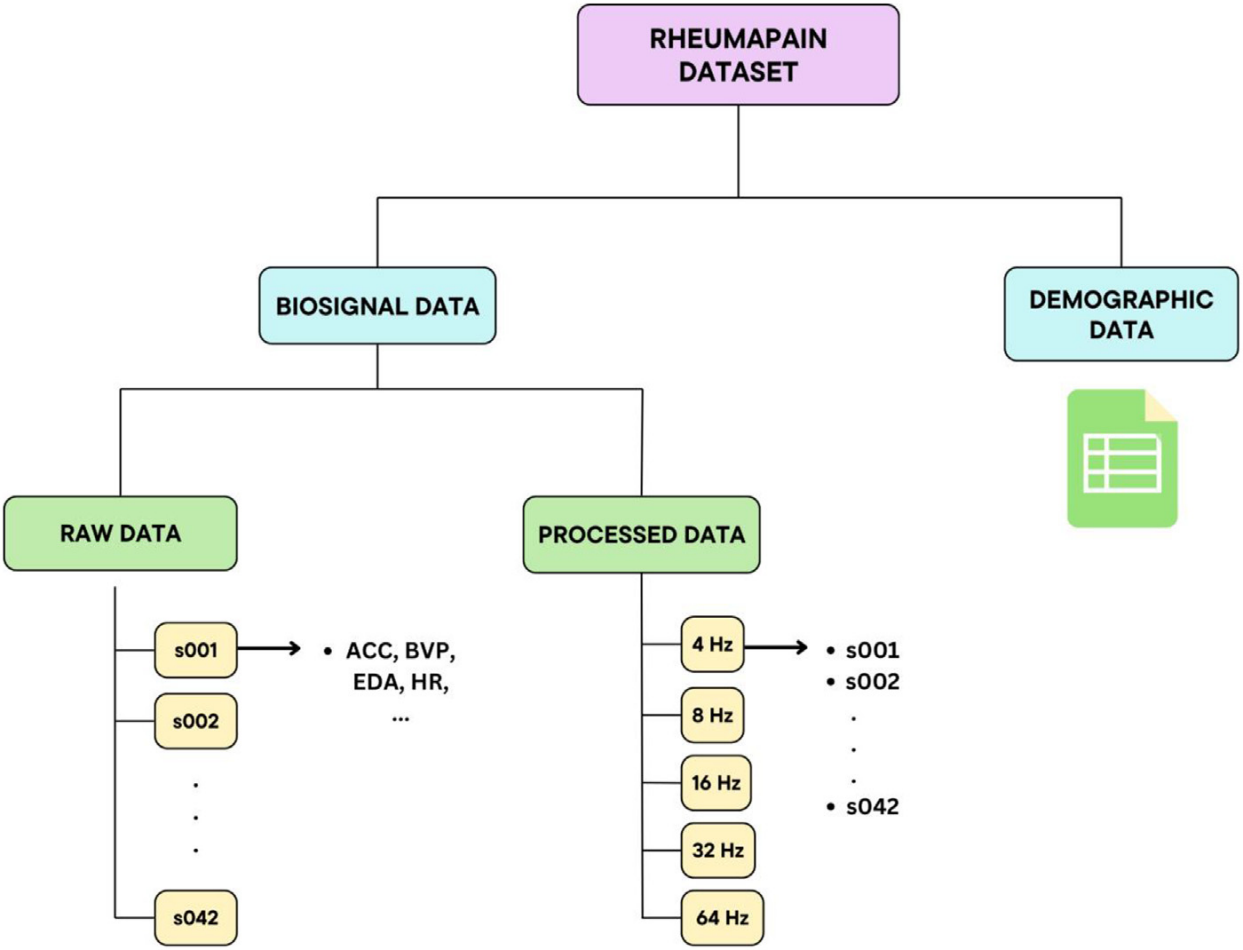
General structure and folder clustering of RheumaPain Dataset.

Fig. 1 depicts the general structure and folder clustering of Rheumapain Dataset. Demographic information, including gender, age, and type of rheumatic disease is stored in the Demographic Data subfolder. Exercise and resting phases were annotated using a stopwatch to ensure accurate phase labeling. The dataset provides both raw and preprocessed biosignal recordings collected from each patient. The raw data include BVP, HR, EDA, ACC, and TEMP signals, which were originally recorded at different sampling frequencies and are stored in the **Raw Biosignal Data** subfolder as separate files for each patient. In the **Preprocessed Biosignal Data** subfolder, signals are presented in synchronized form, where all biosignals are combined into a unified CSV file for each patient together with pain scale scores in resting and exercise time periods, diagnosis information, and patient ID, allowing all relevant information to be analyzed within a single structure. To ensure consistent signal representation, processed CSV files are available at multiple sampling frequencies ranging from 4 Hz to 64 Hz.

In the sections below, the detailed structure of folders, subfolders and files of the dataset are given:

a. Biosignal Data Subfolder: This subfolder contains both raw biosignal and the processed biosignal data of the patients.
  1. Raw Biosignal Data: This subfolder includes data collected from each patient, organized into separate subfolders labeled with each patient’s ID. Each patient folder contains .csv files corresponding to signals, with info, tags, and TEMP files.
  2. Processed Biosignal Data: This subfolder contains the processed biosignal data for each patient. Since each signal type was originally recorded at different sampling rates in the raw data, upsampling and downsampling procedures were applied to synchronize them into a single .csv file per patient. The processed signals are provided at multiple standardized sampling frequencies between 4 and 64 Hz. For each patient, processed data files are available at the following sampling rates: 4 Hz, 8 Hz, 16 Hz, 32 Hz, 64 Hz
b. Demographic Data Subfolder: This subfolder contains patients.xslx file. In this excel sheet, gender, age, pain scale during resting, pain scale during exercise, diagnosis of the patient’s rheumatologic disease, and exercise durations (in seconds) information are given.

Table 1 presents the preprocessed physiological signal data together with detailed descriptions of the recorded body signals, patient id, demographic information, and exercise/rest column to specify whether the patient was performing physical exercise or resting at the time of data acquisition. As pain levels in rheumatology patients commonly increase during physical activity, participants reported different pain scale scores for exercise and resting periods. Therefore, both the pain scale values and the corresponding activity phase (exercise or rest) are provided separately in the dataset. This distinction enables researchers to perform condition-specific analyses and to focus on pain responses in different situations in their studies.

**Table 1:** Detailed information of the columns in RheumaPain dataset.

Data	Data Type	Data Description
**BVP**	float64	Pulsatile waveform acquired using the PPG sensor, reflecting changes in peripheral blood volume. Pulse rate and inter-beat timing features may be derived after appropriate signal-quality assessment.
**EDA**	float64	Skin conductance measured in microsiemens (µS). The signal may vary with sympathetic arousal, physical activity, pain, emotional state, and sensor contact.
**X**	float64	Indicates the acceleration in X-axis.
**Y**	float64	Indicates the acceleration in Y-axis.
**Z**	float64	Indicates the acceleration in Z-axis.
**Temperature**	float64	Indicates the surface temperature of the skin in °C (Celcius). Surface temperature is increased during exercises, it also monitors the patient’s health status.
**Pain Scale**	int64	Includes the answers of the patients to Wong-Baker Faces Pain Scale (0–5).
**Diagnosis**	object	Name of the rheumatic disease of the patient.
**Person_ID**	object	Indicates the ID of the patient.
**Sex**	object	Indicates gender of the patient.
**Age**	int64	Indicates age of the patient.
**Exercise/Rest**	object	Describes if the patient is in exercise or resting phase at that moment.

The resampled files are provided as convenience representations for synchronized multimodal analyses and should not be regarded as equivalent substitutes for the native recordings. Researchers performing signal-specific physiological analyses should use the raw recordings at their native sampling frequencies.

## 4 Experimental Design, Materials and Methods

### 4.1 Research design

**Fig. 2:**
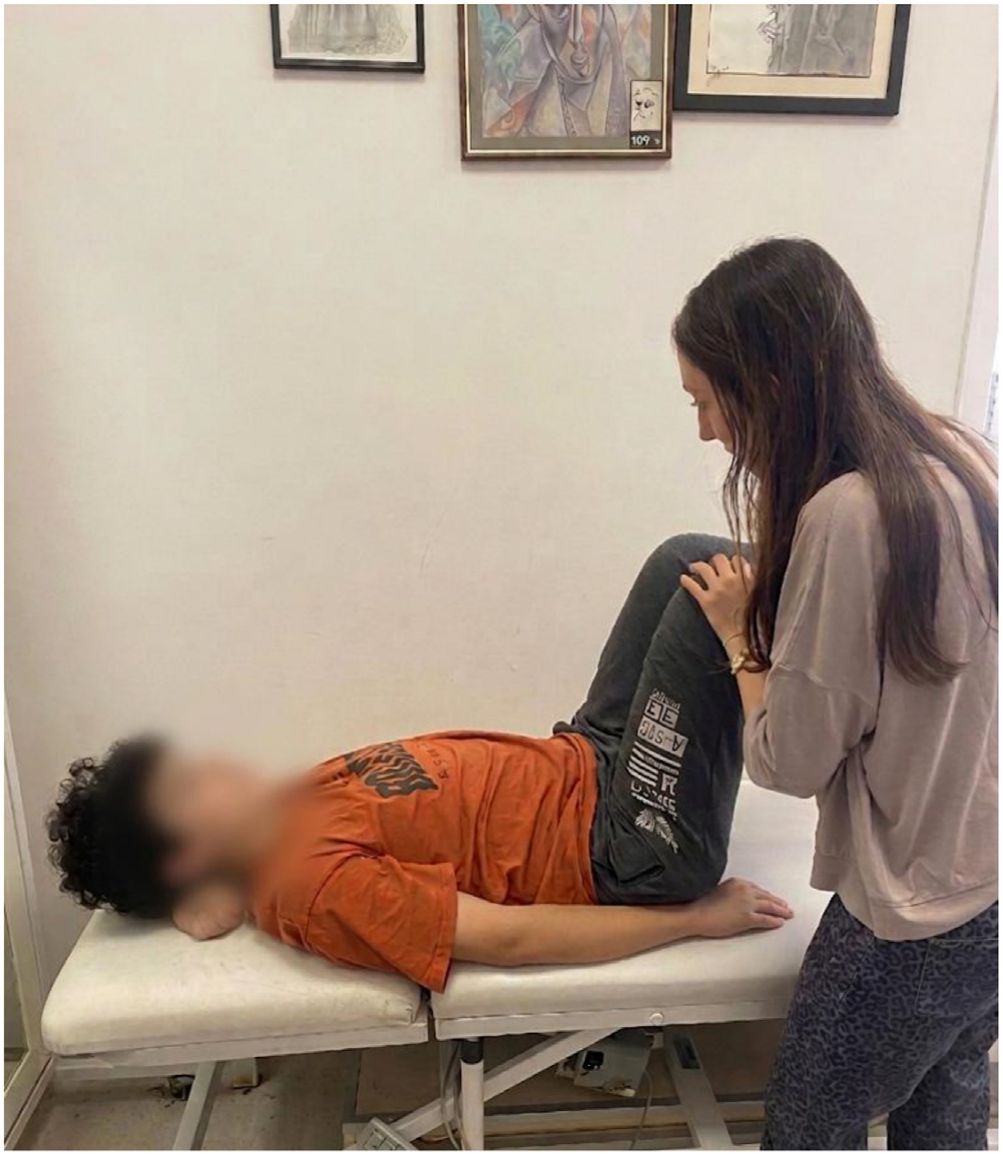
Example of data collection during the exercise phase (face blurred for privacy).

This study is conducted at Istanbul University-Cerrahpaşa, Department of Child Health and Diseases, Department of Pediatric Rheumatology, Physiotherapy and Rehabilitation Unit. The conditions of child patients between the ages of 5–18 suffering from various rheumatological disorders are observed during treatment and at rest. Among the 42 patients included in the dataset, 31 are female and 11 are male. The treatments include the application of a series of exercises recommended to the patients by the faculty members of the Physiotherapy and Rehabilitation Department, and these exercises differ according to the type of various diseases in the field of child rheumatology. The E4 Empatica wearable device is attached to the patients' arms and the physiological signals of the patients are collected with the help of this device. Simultaneously, the patients are asked about the intensity of the pain they felt during exercise and rest using the Wong-Baker Faces Pain Scale [4]. Data is collected in the time periods covering the exercise and rest states. While creating the dataset, these periods were cut and processed by taking into account the time periods when the pain was felt more intensely or less severely. Fig. 2 shows an image of a child rheumatology patient undergoing treatment during exercise.

### 4.2 Wong-Baker faces pain scale

**Fig. 3:**
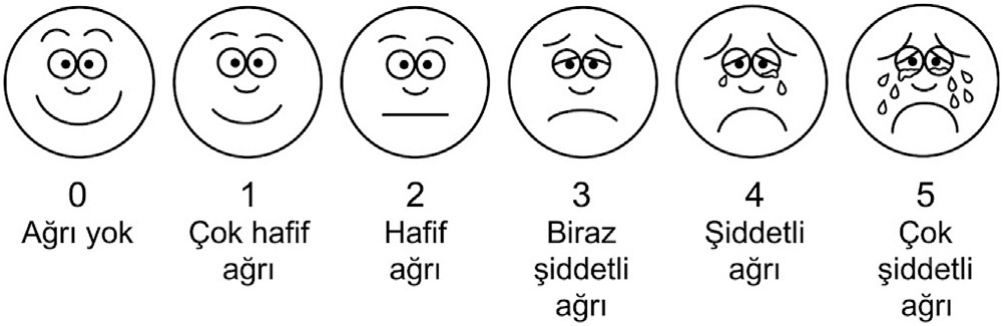
Turkish version of wong-baker faces pain scale.

For the pain scale, with the help of expert physiotherapists, Wong-Baker Faces Pain Scale is used, which is a scale that allows children to express themselves better. Using Wong-Baker FPS, shown in Fig. 3, patients are asked how much pain they felt during both exercise and resting phases. Since this study is conducted in Turkey, the Turkish version of Wong-Baker FPS is used [11].

Although different versions of the FPS exist, the main idea of this scale is to enable young children to express themselves through visual representations, since numbers and words may be difficult for some children in younger age groups to understand. This pain scale is currently used worldwide for children aged three years and older [4].

### 4.3 Signal data

Beside the self-reported pain levels of the patients, their physiological signals are recorded with E4 Empatica Wristband. E4 Empatica has 4 sensing modalities: PPG, EDA, accelerometer and infrared sensor. EDA measures sympathetic nervous system arousal, the accelerometer is 3-axis and captures movement-based activity, and the infrared sensor can track changes in body temperature.

**PPG** is an optical sensing technique used to detect changes in blood volume in the microvascular tissue. In the Empatica E4 wristband, the PPG sensor records the BVP signal, which reflects beat-to-beat changes in blood volume and blood pressure in response to stress stimuli [12]. The PPG sensor operates by emitting light into the skin and measuring changes in the amount of reflected or absorbed light caused by variations in blood volume. The recorded BVP signal can be used to derive HR, heart rate variability (HRV), and related cardiovascular information.

**EDA** reflects changes in skin conductance associated primarily with sympathetic sudomotor activity. EDA is commonly described through tonic skin conductance level and phasic skin conductance responses. For many people, emotional changes, increased cognitive workload, or physical exertion result in the brain sending signals to the skin to increase sweating. Even if no sweat is felt on the skin surface, electrical conductivity increases noticeably as pores below the surface begin to fill [13]. The Empatica E4 provides a way to capture electrical conductivity (the inverse of resistance) in the skin. It achieves this by passing a very small amount of current between two electrodes in contact with the skin. The conductance measurement units are microSiemens (μS). The signal may vary with sympathetic arousal, physical activity, pain, emotional state, and sensor contact. Because EDA may be affected by pain, emotional arousal, physical exertion, movement, and environmental conditions, it should not be interpreted as a pain-specific measure in isolation.

**Fig. 4:**
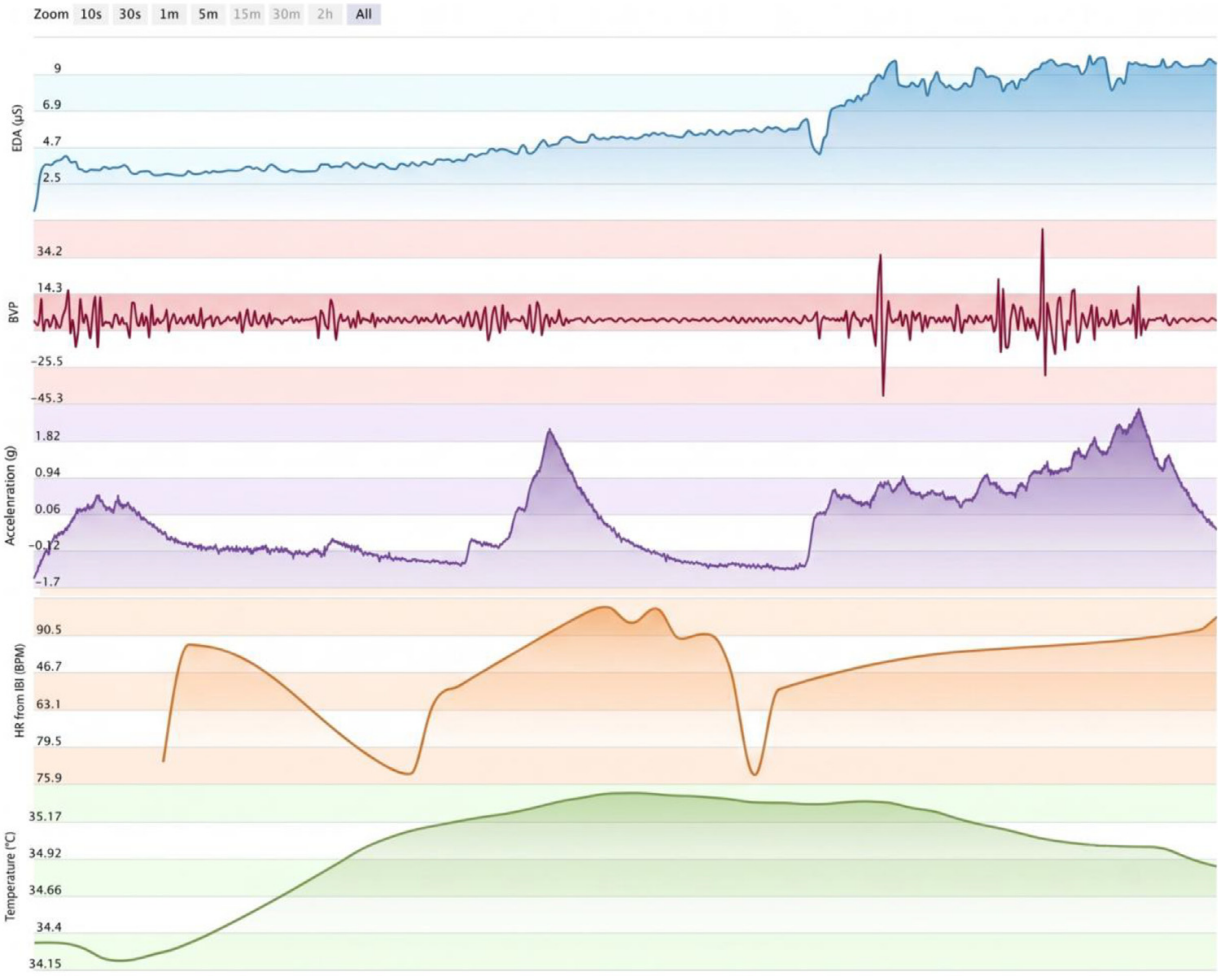
Biosignal data of a 17 years old patient.

Fig. 4 shows the raw biosignal data of a 17-year-old JIA patient obtained from the E4 Empatica cloud platform. Accordingly, at certain moments the heart rate, sweating, and mobility remained relatively stable, while at other moments they accelerated. Exercise and resting periods were also recorded using a stopwatch. However, even by visual inspection of the signals, it is possible to interpret the exercise and rest periods from the data.

### 4.4 Data preprocessing

The native sampling frequencies were 64 Hz for BVP, 32 Hz for triaxial ACC, and 4 Hz for EDA and TEMP. All native recordings are preserved in the Raw Biosignal Data folder. Additional synchronized representations at 4, 8, 16, 32, and 64 Hz are provided to facilitate multimodal data integration. Upsampled versions do not contain additional physiological information beyond the bandwidth of the native signal, whereas downsampled versions may not preserve high-frequency waveform characteristics.

The resampled files are provided to facilitate synchronized multimodal analyses. However, the different resampled representations should not be considered physiologically equivalent. Upsampling does not introduce additional physiological information beyond the native bandwidth of a signal, whereas downsampling may remove waveform information. In particular, BVP representations below the native sampling frequency of 64 Hz are not recommended for pulse-waveform analysis, beat detection, inter-beat interval estimation, or pulse-rate variability analysis. The native 64-Hz BVP recordings should be used for these purposes.

During data collection, the start and end times of the exercise and rest periods were recorded using a stopwatch. Participants reported their pain intensity separately during exercise and rest. These temporal boundaries were mapped onto the synchronized physiological recordings, and the corresponding exercise/rest label and self-reported pain score were assigned to each time segment. Because the exercises and treatment durations differed across participants, the duration and distribution of the labeled exercise and rest periods also varied between participants.

**Fig. 5:**
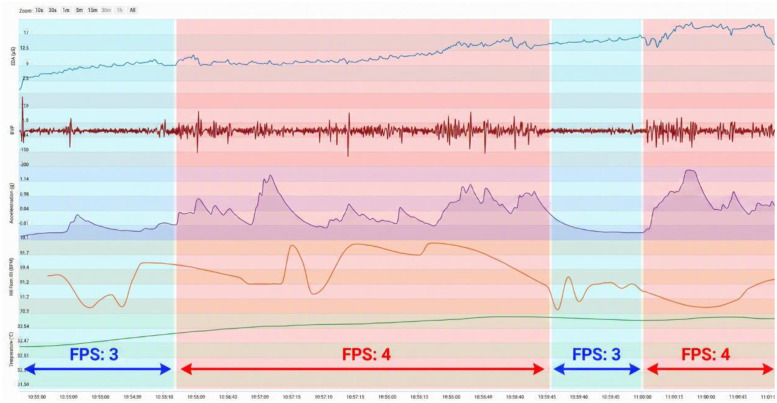
Example of synchronized physiological recordings from an 18-year-old participant diagnosed with juvenile idiopathic arthritis. Blue and red shaded regions indicate rest and exercise periods, respectively, with the corresponding self-reported pain scores.

Table 2 presents the number of samples assigned to each pain level in the five resampled representations of the processed dataset. Fig. 5 illustrates the labeling procedure using data from an 18-year-old female participant diagnosed with juvenile idiopathic arthritis. The participant reported a pain score of 3 during rest and 4 during exercise. Rest periods are shown in blue, whereas exercise periods are shown in red.

**Table 2:** Number of samples assigned to each pain level in the resampled versions of the processed dataset.

Frequency	0	1	2	3	4	5
4Hz	20,444	12,557	22,407	14,303	8461	2010
8Hz	40,906	25,124	44,822	28,584	16,918	4014
16Hz	81,830	50,258	89,650	57,146	33,832	8022
32Hz	163,677	100,526	179,308	114,270	67,660	16,038
64Hz	327,371	201,062	358,622	228,518	135,316	32,070

### 4.5 Statistics of investigation

**Fig. 6:**
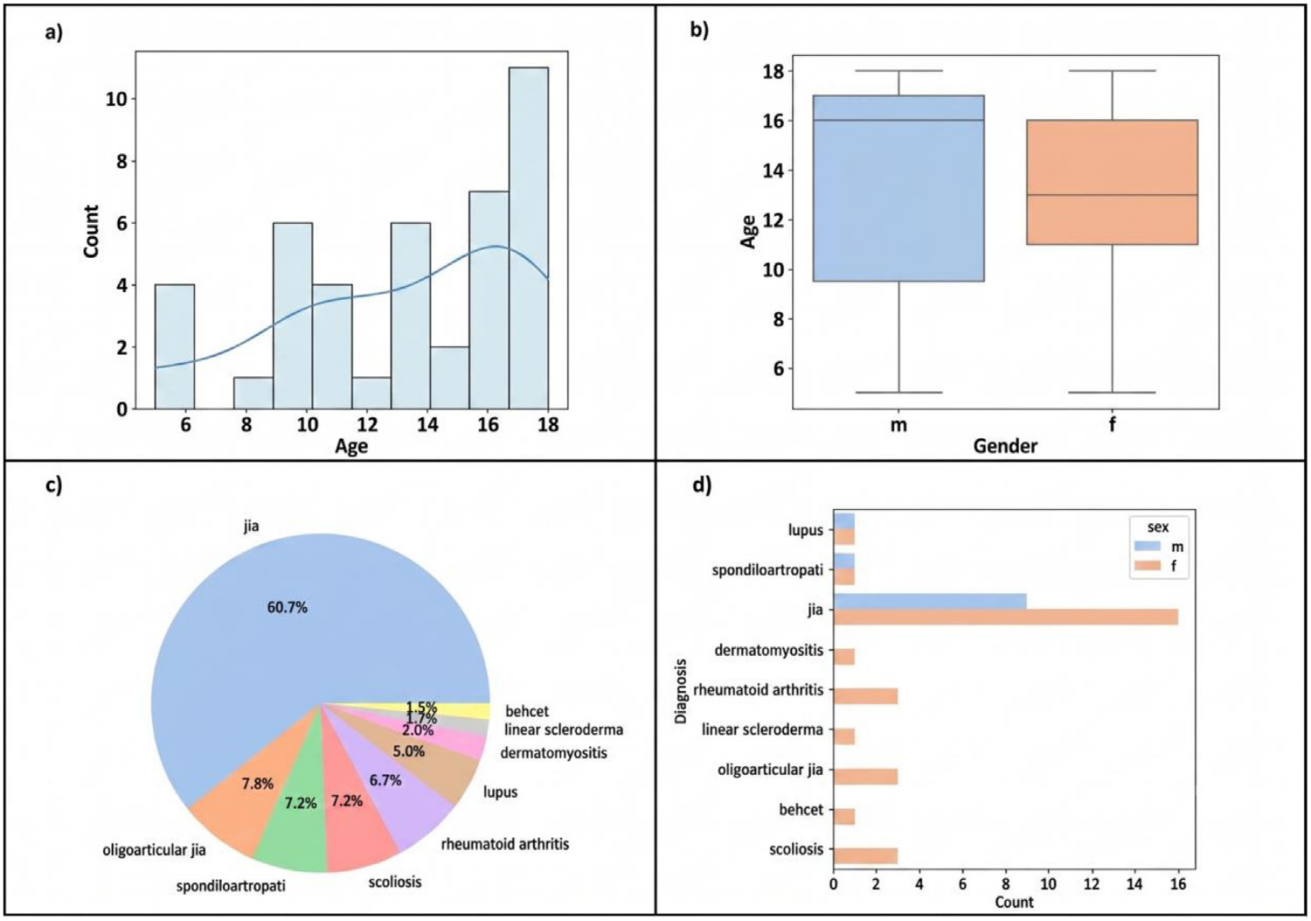
Distribution of diseases according to age and gender.

The population of the study includes 42 children patients suffering from rheumatic diseases aged 13 on average. 11 of them are male while 31 of them are female as it is mentioned in previous sections. In Fig. 6, age and disease classifications are indicated. For this study, it is observed that the most common paediatric rheumatic disease according to the data collected in Cerrahpaşa Hospital is JIA.

**Fig. 7:**
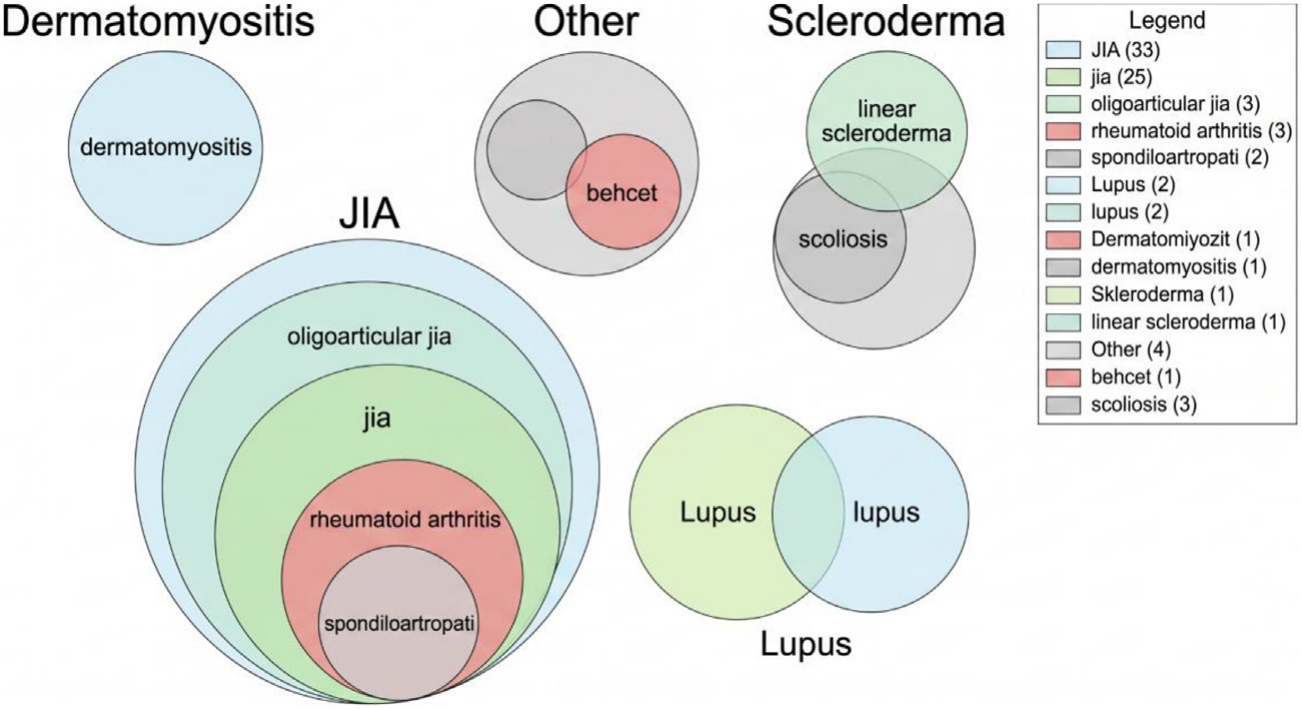
Distribution of rheumatic diseases according to their type.

In Fig. 7, the 5 different groups show the distribution of diseases, which are the four main disease types to define paediatric rheumatic diseases(JIA, JDM, SLE, JSkl) [[14], [15], [16], [17]], and the group named ‘Others’. Oligoarticular JIA, Rheumatoid Arthritis and Spondyloarthropathy and be included in JIA. Lupus, Scleroderma and Dermatomyositis include the diseases named the same with them. The diseases which are not defined in four main groups but still have the characteristics of rheumatic diseases classified in Others group, Behcet and Scoliosis can be given as examples.

**Fig. 8:**
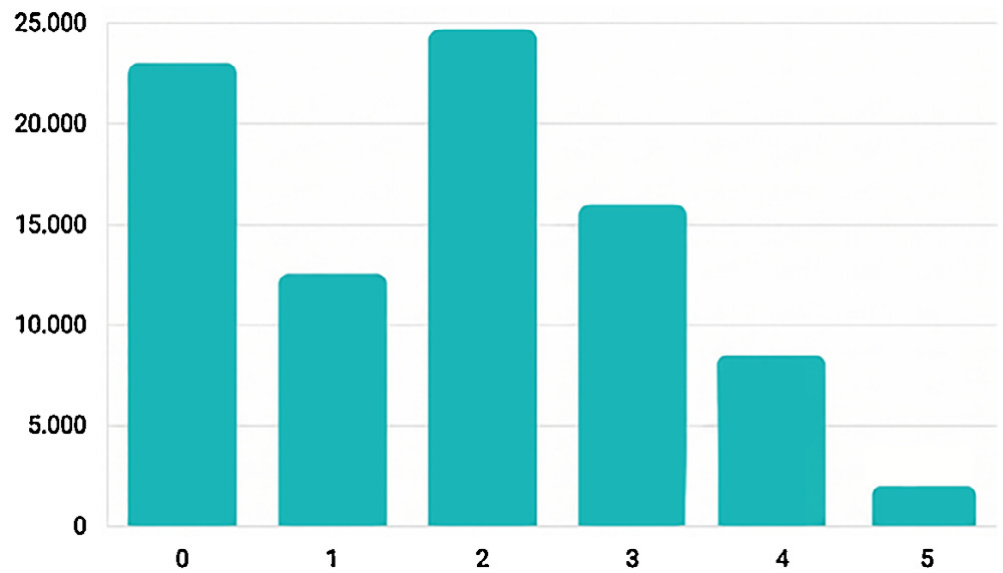
Number of signals collected for each pain level.

In Fig. 8, number of signals that are collected for six levels of pain according to the Wong-Baker Faces Pain Scale are shown. If the collected signals from all patients are considered, the highest samples come from the pain level 2, which takes 25,000 lines for 8 Hz frequency of signal. Least samples come from the pain level 5.

## Limitations

The dataset includes physiological recordings and self-reported pain intensity scores collected during physiotherapy sessions. While a stopwatch was used to record the start and finish times of exercise and rest periods, the physiological onset and reduction of pain may not have coincided precisely with these temporal boundaries. Thus, the pain labels provided are the pain score reported for the respective exercise or rest phase and not the actual day-to-day variation in pain intensity.

When reusing the dataset several pediatric-specific factors also need to be considered. Physiotherapy inherently involves arm and body movement, which may introduce motion artifacts into wrist-based BVP, EDA, ACC, and peripheral skin-temperature recordings. Recordings with persistent signal interruption or evident loss of sensor contact were excluded during the data-quality assessment; however, transient movement-related artifacts may remain in the retained recordings.

The Empatica E4 is not specifically designed or validated for pediatric use. Its adjustable strap provided an adequate fit for most participants; nevertheless, differences in wrist size and movement occasionally affected sensor–skin contact. In addition, participant cooperation and compliance may have varied according to age, discomfort, fatigue, and familiarity with the physiotherapy tasks. The type and duration of exercise were not identical across participants because the physiotherapy sessions were determined according to their clinical conditions. These differences should be considered when comparing participants or diagnostic groups.

Self-reported pain assessment in children may also be influenced by age, cognitive development, previous pain experience, and interpretation of the facial expressions presented on the Wong–Baker Faces Pain Scale. The scale was administered with the support of the supervising physiotherapist; however, the recorded pain label represents the participant’s self-reported score. The physiotherapist’s observations were used only to support the interpretation of the reported scores and were not used to replace or numerically modify them. These factors may introduce label uncertainty and should be considered when developing and evaluating predictive models based on the dataset.

## CRediT Author Statement

**Elif Yıldırım:** Data Collection, Modelling, Data curation, Writing - Original Draft; **Fatma Patlar Akbulut**: Conceptualization, Supervision, Methodology, Validation, Writing - Review & Editing.

## Ethics Statement

For this study, data were collected with the formal approval of the Ethics Committee of Istanbul Kultur University (dated 14.10.2025 and numbered 180,853).

## Data Availability

Mendeley DataRheumaPain Dataset (Original data)
